# Regorafenib-Triggered Hemophagocytic Lymphohistiocytosis: A Report of Two Cases

**DOI:** 10.7759/cureus.72721

**Published:** 2024-10-30

**Authors:** Ilfad Blazevic, Nadim Fares, Blandine Delaunay, Carlos Gomez-Roca

**Affiliations:** 1 Department of Medical Oncology, Oncopole Claudius Regaud, Toulouse, FRA; 2 Department of Digestive Oncology, Toulouse University Hospital, Toulouse, FRA

**Keywords:** breast cancer, colorectal cancer, fever with rash, hemophagocytic lymphohistiocytosis (hlh), high ferritin, regorafenib

## Abstract

Hemophagocytic lymphohistiocytosis (HLH) is a rare, potentially fatal condition, characterized by overactivation of the immune system. It can manifest as primary or secondary when triggered by infections, neoplasms, or medications. We report here two cases of regorafenib-induced HLH, one of which was life-threatening. The first patient was a 67-year-old woman with metastatic triple-negative breast cancer; the second patient was a 64-year-old woman with metastatic colon cancer. Both patients were included in the REGOMUNE clinical trial, a phase I/II trial evaluating the combination of regorafenib with avelumab. In this trial, the first administration of avelumab occurred on the 15th day of the cycle. Both patients developed a febrile rash: on the 10th day of regorafenib treatment for the first patient and on the 12th day for the second. Elevated levels of ferritin, C-reactive protein, and thrombocytopenia were observed in both cases. Bone marrow aspiration revealed hemophagocytosis, leading to the diagnosis of HLH for the two patients. The second patient had a more severe form with rapid hemodynamic deterioration, requiring transfer to the intensive care unit. The outcome was favorable in both cases after the definitive discontinuation of regorafenib and initiation of corticosteroid therapy. HLH is a rare adverse effect of regorafenib that can be life-threatening. Therefore, clinicians should promptly consider this diagnosis when encountering manifestations consistent with HLH to ensure timely and appropriate management.

## Introduction

Regorafenib is an orally bioavailable multi-kinase inhibitor, which has activity against several targets including vascular endothelial growth factor receptor (VEGFR), fibroblast growth factor receptor (FGFR), and platelet-derived growth factor receptor (PDGFR) [1]. It is currently approved for the treatment of metastatic colorectal cancer, unresectable hepatocellular carcinoma, and advanced gastrointestinal stromal tumor. Regorafenib causes also a modulation of the tumoral microenvironment, especially via macrophages, resulting in an enhancement of the antitumoral immunity [2-5]. Due to these immunomodulating properties, regorafenib has been and is currently evaluated in combination with programmed death (PD-1) and programmed death 1 ligand (PD-L1) inhibitors [6-8]. Thus, this treatment combination can be considered for patients who have no other therapeutic alternatives.

Hemophagocytic lymphohistiocytosis (HLH) is a rare life-threatening condition of hyperinflammation caused by activated macrophage proliferation. Clinical and laboratory findings include fever, hepatosplenomegaly, cytopenias, hypertriglyceridemia, hemophagocytosis, and elevated ferritin. There are two types of HLH: familial HLH (FLH), which is linked to an underlying genetic abnormality, involving genes such as *perforin-1*, and secondary HLH, which is associated with an infection, a metabolic disorder, a malignancy, or an autoimmune disease [9]. In addition, several cases of drug-induced HLH have been reported in the literature: lamotrigine, carbamazepine, dabrafenib, and trametinib [10-12]. To our knowledge, only one case of regorafenib-induced HLH was previously reported [13].

We describe two new cases of an HLH that occurred during regorafenib treatment and were successfully managed with treatment discontinuation and corticosteroid therapy.

## Case presentation

Case 1

The first patient was a 67-year-old woman with a past medical history of hypertension and total thyroidectomy. She was diagnosed in May 2017 with a left T2N1M0 grade 2 apocrine triple-negative breast carcinoma and received neoadjuvant chemotherapy with epirubicin plus cyclophosphamide and paclitaxel, followed by a radical mastectomy plus axillary clearance and adjuvant radiotherapy. The patient developed in May 2022 a locoregional and pulmonary recurrence confirmed with a biopsy. She was treated with a combination of carboplatin, gemcitabine, and pembrolizumab allowing a metabolic partial response. In February 2023, she presented with a pulmonary and mediastinal progression, which led to her inclusion in the REGOMUNE phase I/II (NCT03475953) clinical trial evaluating the combination of regorafenib with avelumab in solid tumors [7]. In this trial, regorafenib was planned to be taken daily at 160 mg for three weeks of a four-week cycle. The first administration of avelumab was planned on day 15 of the first cycle and on days one and 15 of the subsequent cycles. The patient started regorafenib as a single agent on May 11, 2023.

**Figure 1:**
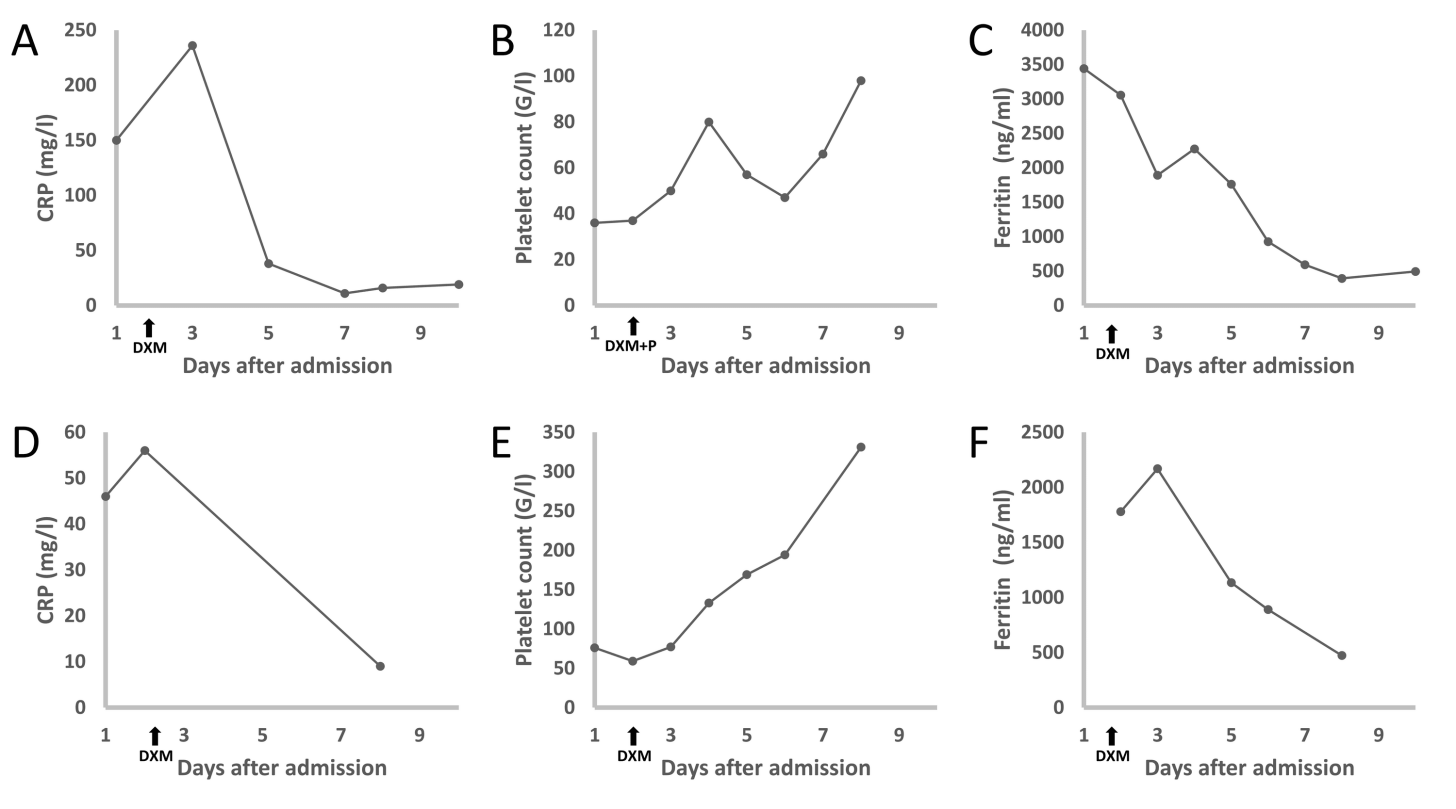
Evolution of C-reactive protein (CRP), platelet count, and ferritin for patient 1 (A, B, and C) and patient 2 (D, E, and F). DXM: dexamethasone administration, P: platelet transfusion

The patient was admitted to our institute on May 23, 2023 (representing day one) due to a 40°C fever and a cutaneous rash that had started two days earlier. The clinical examination revealed a maculopapular rash on the limbs and the trunk, without organomegaly, hypotension, or tachycardia. Laboratory exams showed a hemoglobin level of 12.4 g/dL, neutrophil count of 4.2 G/L, platelet count of 36 G/L, serum creatinine of 138 µmol/L, alanine aminotransferase (ALAT) of 42 U/L, aspartate aminotransferase (ASAT) of 71 U/L, total bilirubin of 15 µmol/L, C-reactive protein (CRP) of 150 mg/L, ferritin of 3442 ng/mL, prothrombin time (PT) of 75%, fibrinogen of 3.9 g/L, and triglyceride of 1.3 mmol/L. Regorafenib was discontinued with a last intake on May 22, 2023. Due to the suspicion of an HLH, a bone marrow aspiration was done on day two of admission, showing histiocytes with features of hemophagocytosis of the platelets. The HScore assesses the probability of HLH based on clinical and biological parameters such as fever, hepatosplenomegaly, platelet count, and ferritin concentration [14]. In this patient, HScore was 138, indicating a 13.8% probability of HLH. She had negative viral polymerase chain reaction (PCR) results in the blood and marrow, including Epstein-Barr virus, cytomegalovirus, parvovirus B19, herpes simplex virus 1 and 2, human herpesvirus-6, varicella-zoster virus, and hepatitis A, B, C, and E viruses. Blood cultures remained negative. A skin biopsy revealed a lymphohistiocytic and eosinophilic infiltrate. The patient received a short-course dexamethasone treatment with a 10 mg injection on day two and two additional 10 mg injections on day three. Figure 1 provides an overview of the biological parameters during hospitalization. Following the administration of dexamethasone, there was a rapid decrease in temperature, ferritin, and CRP levels. Concurrently, there was a gradual improvement in the platelet count and maculopapular rash. No probabilistic antibiotic therapy was initiated. Renal ultrasound was performed and was considered normal without signs of obstruction. Renal function returned to normal after intravenous rehydration. The final diagnosis of HLH was retained. Regorafenib was permanently discontinued and the patient was withdrawn from the clinical trial. The disease remained stable according to Response Evaluation Criteria in Solid Tumors (RECIST, version 1.1) on the last CT scan available in February 27, 2024. The patient is doing well, presents a stable disease, and is under simple surveillance.

Case 2

The second patient was a 64-year-old woman with no significant medical history who was diagnosed with colonic adenocarcinoma at the age of 61. She was treated with a colectomy followed by adjuvant 5-fluorouracil/leucovorin oxaliplatin (FOLFOX) chemotherapy. The patient presented with a peritoneal recurrence treated with 5-fluorouracil/leucovorin irinotecan (FOLFIRI) bevacizumab in July 2022, which was interrupted due to poor tolerance. After progression, our patient started Nivolumab in December 2022 due to the finding of a high tumor mutational burden (36.9 mutations per megabase). In July 2023, she underwent complete cytoreduction and hyperthermic intraperitoneal chemotherapy. Lymph node and pleural recurrence occurred in January 2024, leading to her inclusion in the REGOMUNE trial [7]. The patient started Regorafenib on January 23, 2024.

On February 3, 2024, after 12 days of treatment, the patient presented with a rash, fever, and dyspnea. She consulted the emergency department on February 5, 2024, which represents day one of admission in the present case. Regorafenib was discontinued at that time. A chest CT scan revealed no pulmonary embolism or infection. Laboratory tests showed a hemoglobin level of 14.3 g/dL, neutrophil count of 5.2 G/L, thrombocytopenia at 76 G/L, serum creatinine of 68 µmol/L, ALAT of 61 U/L, ASAT of 64 U/L, gamma-glutamyltransferase (GGT) of 27 U/L, total bilirubin of 22.9 µmol/L, CRP of 46 mg/L, and sodium of 127 mmol/L. Ferritin was measured at 1778 µg/L. The patient rapidly experienced hemodynamic deterioration, with arterial hypotension of 80/60 mmHg requiring vascular expansion with saline solution. Broad-spectrum antibiotics were initiated following blood and urine cultures. Due to persistent hemodynamic instability, the patient was transferred to the intensive care unit where norepinephrine was administered. Given the suspicion of HLH, a myelogram was performed, revealing a few activated macrophages with rare hemophagocytosis images. The HScore was 157, corresponding to a 34.2% probability of HLH [14]. Intravenous corticosteroid therapy with dexamethasone at a dose of 10 mg every eight hours was initiated on February 6, 2024, leading to a rapid reduction in fever and gradual improvement of the rash and thrombocytopenia. The bacteriological assessment was negative, allowing for the cessation of antibiotic therapy after five days. Dexamethasone was also discontinued on day five of admission without recurrence of fever or rash in the following days. The final diagnosis of HLH was retained. Regorafenib was discontinued, but the patient remained in the study for follow-up. The patient was discharged after a few days of monitoring in our department. The latest oncological evaluation on March 14, 2024, showed stable disease.

## Discussion

Similar to the previously reported case of regorafenib-induced HLH in the literature [13], these two patients showed fever, asthenia, and rash without hepatosplenomegaly or adenomegaly as presenting symptoms. Both patients also exhibited laboratory similarities, including decreased platelet count and moderate ferritin elevation. In the present manuscript, the second patient had a more severe form of HLH, with hypotension requiring transient intensive care therapy. Both cases improved promptly after regorafenib discontinuation and a short-course corticosteroid therapy.

The reasons why some patients develop HLH while on regorafenib remain unclear. One potential explanation can be alterations in the *perforin-1 *(*PRF-1*) gene. Perforin is a protein involved in immune modulation and apoptosis. It is released by cytotoxic cells and creates channels in the membrane of the target cell, allowing extracellular fluid to enter and triggering apoptosis. Mutations in *PRF-1*​​ and related genes have been implicated in the development of a majority of FLH [9]. Al-Samkari et al. reported a case of a woman who developed severe HLH while being treated with pembrolizumab for aggressive metastatic breast cancer [15]. Next-generation sequencing revealed a germline polymorphism in the *PRF-1 *gene, suggesting a predisposing factor for HLH, with pembrolizumab acting as a trigger through its immunomodulation effect. Cetica et al. examined biological samples from a large registry of 500 Italian patients with HLH [16]. In this cohort, of the 281 (56%) patients who had a sporadic HLH, 43 had heterozygous mutations in one of the FLH-related genes, such as *PRF-1* or *UNC13D*. The authors propose three forms of HLH: secondary HLH, where no mutation in FLH genes exists and a strong trigger such as a *Leishmania *infection drives the condition; sporadic HLH, when the patient has a monoallelic mutation in an FLH-related gene where the trigger plays a moderate role; and genetic HLH, where biallelic mutations in FLH-defining genes can cause HLH with no or mild triggers like commonly encountered viral pathogen. No genetic testing was performed on the patients in this case report. Both primary and secondary forms of HLH show histologic features of hemophagocytosis which is due to dysregulation of the immune system. Besides its tyrosine kinase inhibition activity, regorafenib has various effects on the immune cells: it increases T cell proliferation and activation and increases the ratio of M1/M2 polarized macrophages [5]. These immunological effects of regorafenib may explain the onset of HLH in predisposed cancer patients, potentially contributing to prolonged disease control, as observed in the first patient.

## Conclusions

HLH is a rare adverse effect of regorafenib, which can be life-threatening as illustrated in the present case reports. In the presence of clinical and laboratory findings such as fever, rash, cytopenias, and elevated ferritin levels, the syndrome should be promptly identified by clinicians due to the risk of clinical deterioration. Appropriate management should be initiated, including the treatment of any potential organ failures, discontinuation of regorafenib, and corticosteroid therapy. The pathophysiology of regorafenib-induced HLH remains unclear; however, immune system modulation, particularly of macrophages in patients with predisposing factors, appears to be a plausible explanation.
